# Estradiol Exposure Differentially Alters Monolayer versus Microtissue MCF-7 Human Breast Carcinoma Cultures

**DOI:** 10.1371/journal.pone.0157997

**Published:** 2016-07-05

**Authors:** Marguerite M. Vantangoli, Samantha J. Madnick, Shelby Wilson, Kim Boekelheide

**Affiliations:** Department of Pathology and Laboratory Medicine, Brown University, 70 Ship Street, Providence RI 02903, United States of America; University of South Alabama Mitchell Cancer Institute, UNITED STATES

## Abstract

The development of three-dimensional (3D) cultures is increasing, as they are able to provide the utility of *in vitro* models and the strength of testing in physiologically relevant systems. When cultured in a scaffold-free agarose hydrogel system, MCF-7 human breast carcinoma cells organize and develop into microtissues that contain a luminal space, in stark contrast to the flat morphology of MCF-7 two-dimensional (2D) monolayer cultures. Following exposure to 1nM E2, expression of typical estrogen-responsive genes, including progesterone receptor (PGR), PDZ containing domain 1 (PDZK1) and amphiregulin (AREG) is increased in both 2D and 3D cultures. When examining expression of other genes, particularly those involved in cell adhesion, there were large changes in 3D MCF-7 microtissues, with little to no change observed in the MCF-7 monolayer cultures. Together, these results indicate that while the initial estrogen-regulated transcriptional targets respond similarly in 2D and 3D cultures, there are large differences in activation of other pathways related to cell-cell interactions.

## Introduction

As a result of the National Research Council report “Toxicity Testing in the 21^st^ Century: A Vision and a Strategy,” [1] there is a large need to develop more efficient and physiologically relevant models for evaluation of compound safety and toxicity. Due to the cost- and time-intensive nature of animal testing, *in vitro* based screening models are growing in favor. Additionally, *in vitro* cell culture models allow for the use of human cell lines, enabling researchers to better evaluate human health and disease states when compared to *in vivo* animal models.

Traditionally, human primary cells and cell lines have been cultured in 2-dimensional (2D) monolayer on plastic substrates. While 2D cultures are technically easy to conduct and inexpensive, they do not recapitulate the biology of cells *in vivo*, where tissues are highly cell dense and cells are in contact with other cell types and extracellular matrix (ECM). Previous work has demonstrated that this contact with ECM and cells can alter gene expression profiles and cellular morphology [2–5], potentially leading to differences in experimental results between 2D *in vitro* and intact *in vivo* systems.

As an alternative strategy to address several of the shortcomings of both *in vivo* and 2D *in vitro* experimental models, 3-dimensional (3D) culture models have been developed. A variety of models exist, with two of the most popular models being scaffold-based and scaffold-free systems. In scaffold-based 3D culture, cells of interest are seeded at low density within natural or synthetic matrices. When cultured in Matrigel, a laminin-rich matrix, both normal and malignant human breast cells form 3D cultures with morphologies dependent on their gene expression profiles [3]. Nonmalignant MCF-10A cells cultured in Matrigel have been demonstrated to form lumen-containing mammary acini through apoptosis, a structure reminiscent of the *in vivo* morphology of the adult mammary gland [6]. When transformed into malignant cells, MCF-10A cells become invasive, distorting the structure and morphology of the *in vitro* mammary acini in a process that promotes oncogenesis [6–8]. This and similar systems have provided important insight into the process of both morphogenesis and oncogenesis; however, these models are not without limitations. Typically, cells are seeded embedded within matrix at low densities that do not mimic the highly cellular nature of epithelial tissues *in vivo*. Scaffold-based models are highly dependent on the nature of the scaffold material, as previous work has shown that different matrices including collagen I and laminin can have distinct effects on cells, including altering their invasive potential and gene expression profiles [9–12]. Many scaffold materials do not accurately reconstruct the in *vivo* ECM content, and often rely on only one component such as collagen, while ignoring the heterogeneous nature of native ECM.

Scaffold-free culture models including hanging drop and non-adhesive agarose hydrogels allow for cells to assemble into 3D microtissues free from the influence of outside matrix. While the hanging drop method requires no specialized equipment to form 3D cultures, it is technically difficult to change media and the low media to cell ratio increases the rate of waste accumulation, making long-term cultures difficult to assess. Additionally, hanging drop cultures are susceptible to being physically disturbed, and this can lead to a loss of samples through handling. The use of nonadhesive agarose hydrogels to culture cells in a scaffold-free environment circumvents these problems, as the system allows for easy media changes and handling of cultures. In this system, cells are trypsinized and seeded into non-adhesive hydrogels, where they settle into recesses and come into contact with other cells [13]. Within 24 hours after seeding, cells self-assemble into microtissues free of the influence of outside matrix and substrates [14]. Previous work using MCF-7 cells has demonstrated that when cultured in this model, MCF-7 cells form 3D microtissues that are more differentiated than 2D cultures, have apical-basal polarity and contain a luminal space, reminiscent of the *in vivo* morphology of the adult mammary gland [15].

In the present study, we describe differences in gene expression between 2D monolayer and 3D MCF-7 microtissue cultures following exposure to estradiol (E2). These results indicate that E2 treatment alters expression of classical estrogen responsive genes in a similar matter in both 2D and 3D cultures. However, with longer exposure to E2, the similarities of the response in both culture systems subside and the responses diverge.

## Materials and Methods

### Chemicals and Reagents

Cell culture media and supplements were purchased from Life Technologies, Inc (Grand Island, NY). Fetal bovine serum (FBS) was purchased from Atlanta Biologicals (Flowery Branch, GA) and dextran-coated-charcoal (DCC) stripped was purchased from Gemini Bioscience (Sacramento, CA). Estradiol (E2), tamoxifen (TAM), dimethylsulfoxide (DMSO), Trizol and insulin were purchased from Sigma Aldrich (St. Louis, MO). Agarose was purchased from Fisher Scientific (Agawam, MA).

### Cell Culture

MCF-7 cells (HTB-22) [16] were purchased from ATCC (Manassas, VA) and maintained in DMEM-F12 complete media supplemented with 10% fetal bovine serum (FBS), MEM nonessential amino acids, gentamicin and 10μg/mL insulin in a 5%CO_2_ incubator at 37°C as previously described [15]. Media was changed every 2–3 days and cells were passaged when 65–80% confluent. MCF-7 cells were limited to use within the first 10 passages from the original purchased vial from ATCC, to control for genomic drift due to instability.

### 3D Scaffold-Free Cell Culture

MCF-7 cells were seeded into agarose hydrogels prepared as previously described [15, 17] at a density of 600,000 cells/mL. Cells were allowed to settle into wells for 30 minutes and then placed in 2mL complete growth media, with media changes every 2–3 days until estrogen treatment.

### Estrogen Treatment of MCF-7 Cultures

For monolayer cultures, MCF-7 cells were seeded at a density of 300,000 cells/mL in 6-well plates, and allowed to grow for 3 days in complete media. Cells were then washed twice with PBS and placed in phenol red-free DMEMF12 media supplemented with 5% dextran-charcoal stripped fetal bovine serum, MEM nonessential amino acids, gentamicin and 6ng/mL bovine insulin for 48 hours. For 3D cultures, cells were seeded into agarose hydrogels as above and grown for 7 days in 10% complete media, with media changed every 2–3 days. After 7 days of growth in complete media, 3D MCF-7 cultures were washed twice with PBS and transferred to fresh treatment media for 48 hours. Following 48 hours, 2D and 3D cultures were exposed to estradiol or vehicle control (dimethylsulfoxide) for 8 hours and collected in Trizol for gene expression analysis. All experiments were performed in triplicate.

### RNA Isolation, RT-PCR and PCR Array Analysis

Monolayer cultures were collected by scraping into Trizol. Microtissues were collected from hydrogels by centrifugation, pelleted and lysed in Trizol. Samples were then isolated using the RNEasy Mini Kit (Qiagen) per manufacturer’s instructions. RNA quantity was determined using a Nanodrop ND1000, and quality was determined using an Agilent 2100 Bioanalyzer. A RIN value of greater than 0.7 was used as a cutoff for further analysis using PCR arrays.

A previously described [15] custom SABiosciences PCR Array (Qiagen) was created to include a list of 84 genes generated from literature to evaluate estrogen receptor signaling, breast and ductal morphogenesis, cellular growth and differentiation, proliferation, tumor progression and epithelial to mesenchymal transition as well as 5 housekeeping genes (S1 Table). Genomic DNA contamination and reverse transcription controls were included on the plate, and genomic controls showed no amplification. Samples were prepared per manufacturer’s instructions, and were added to 384-well plates using an epMotion 5075 automated pipettor (Eppendorf). Plates were run on an ABI 7900HT machine using cycling conditions recommended by the manufacturer.

### Statistical Analysis

The raw PCR cycle (Ct) values were imported into the R statistical environment [18]. Raw PCR cycles were normalized (dCt) using the SLqPCR package [19] to optimize selection of house-keeping genes.

For comparison of E2 treated samples to respective vehicle controls and comparison of normalized E2-treated 2D and 3D samples, dCt were used to construct a linear model of adjusted Ct values using the LIMMA package in R [20]. The empirical Bayes statistic was used and genes with an adjusted p-value significance for multiple comparisons (q-value) or less than 0.05 and absolute fold chance of greater then 1.5 were selected.

### Cluster Analysis

Fold change values for all significantly changed genes across time and culture method were log transformed and imported into Cluster [21], and clustered using Euclidean distance and complete linkage analysis. Values were imported into Treeview (reference) and visualized.

## Results

When compared to vehicle-treated controls, MCF-7 monolayer cultures exposed to 1nM estradiol (E2) for 4 hours exhibit significant changes in expression of 19 transcripts as summarized in Table 1. E2 increased expression of insulin-like growth factor binding protein 4 (IGFBP4), progesterone receptor (PGR), V-Myc avian myelocytomatosis viral oncogene homolog (MYC), transforming growth factor alpha (TGFA) and FBJ murine osteosarcoma viral oncogene homolog (FOS). Treatment of MCF-7 monolayer cultures with E2 for 4 hours also increased expression of growth response to estrogen breast cancer (GREB1), PDZ-domain containing 1 (PDZK1), cyclin D1 (CCND1), snail family zinc finger 1 (SNAI1), transforming growth factor beta 3 (TGFB3) and xbox-binding protein 1 (XBP1). E2 treatment also increased expression of vascular endothelial growth factor alpha (VEGFA), amphiregulin (AREG), and wingless-type MMTV integration site family member 4 (WNT4). Expression of BCL2-like 1 (BCL2L1), Erb-B2 receptor tyrosine kinase 2 (ERBB2twist family transcription factor 1 (TWIST1), g-protein coupled estrogen receptor (GPER) and cytochrome P450 1A1, (CYP1A1) were significantly decreased following exposure to estradiol. Complete gene expression results are reported in S2 Table.

**Table 1:** 

Gene Name	Abbreviation	Fold Change	q value
Insulin-Like Growth Factor Binding Protein 4	IGFBP4	5.78	<0.0001
Progesterone Receptor	PGR	4.59	<0.0001
V-Myc Avian Myelocytomatosis Viral Oncogene Homolog	MYC	3.72	<0.0001
Transforming growth factor, alpha	TGFA	3.61	0.0001
FBJ Murine Osteosarcoma Viral Oncogene Homolog	FOS	3.35	0.0001
Growth response to estrogen, breast cancer 1	GREB1	3.23	0.0001
PDZ domain containing 1	PDZK1	3.17	<0.0001
Cyclin D1	CCND1	2.75	0.0002
Snail Family Zinc Finger 1,	SNAI1	2.60	0.0052
Tranforming growth factor beta 3	TGFB3	2.50	0.0004
Xbox binding protein 1	XBP1	2.10	0.0011
vascular endotheilal growth factor alpha	VEGFA	2.01	0.0089
Amphiregulin	AREG	1.84	0.0207
Wingless-Type MMTV Integration Site Family, Member 4	WNT4	1.68	0.0123
BCL2-like 1	BCL2L1	-1.58	0.0306
Erb-B2 Receptor Tyrosine Kinase 2	ERBB2	-1.78	0.0454
Twist Family BHLH Transcription Factor 1	TWIST1	-2.10	0.0023
G-protein coupled estrogen receptor	GPER	-3.06	<0.0001
cytochrome P450 1A1	CYP1A1	-3.27	<0.0001

**Fig 1:**
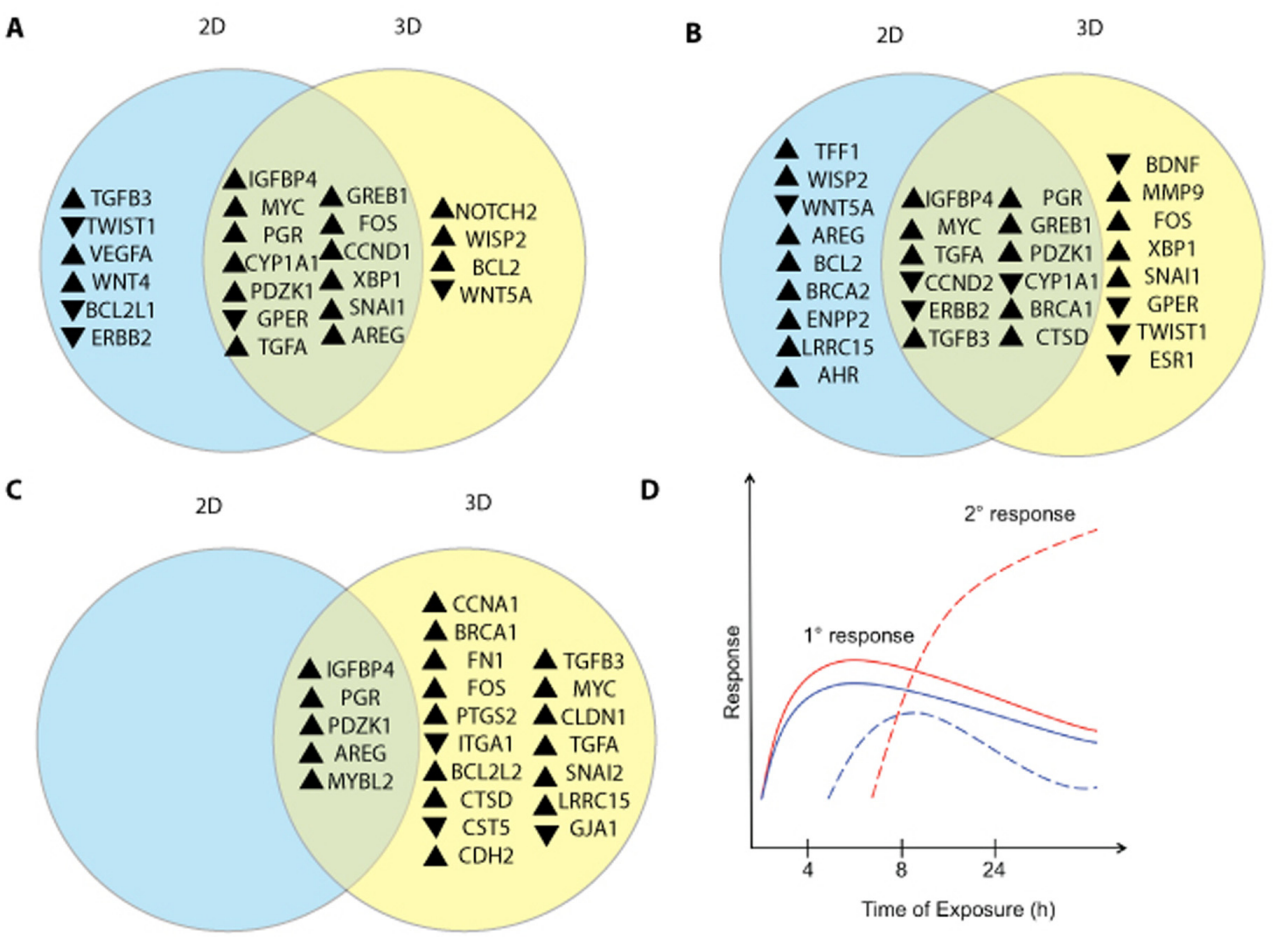
Gene expression of 2D and 3D cultures exposed to 1nM E2 for 4, 8 and 24 hours. MCF-7 2D and 3D cultures were treated with 1nM E2 for 4 hours and E2 treatment of 2D cultures altered expression of 19 transcripts (blue), 14 of which were also altered in 3D cultures (A). Four transcripts were significantly altered in 3D microtissues and not in monolayer cultures (yellow) (A). Monolayer MCF-7 cultures treated with E2 for 8 hours exhibited significant gene expression changes in in 21 transcripts (blue), while 3D microtissues treated with E2 display altered expression of 20 transcripts (yellow) (B). Twelve transcripts were significantly altered in both 2D and 3D cultures (B). E2 treatment altered expression of 5 transcripts in 2D cultures (blue), while 3D cultures exhibited altered expression of 22 transcripts (yellow) following 24 hours of estradiol exposure (C). Five transcripts were significantly altered in both 2D and 3D cultures (C). Responses in 2D and 3D MCF-7 culture differ following exposure to estradiol. In both 2D and 3D cultures, estrogenic responses to estradiol are similar, and involve altered expression of typical estrogen-responsive genes (D). At 8 hours of exposure, secondary responses diverge in 2D and 3D systems, with differences persisting at 24 hours (D).

Exposure of 3D MCF-7 microtissues to estradiol for 4 hours altered expression of 17 transcripts (Table 2), resulting in an increase in expression of PGR, WNT1-inducible signaling protein 2 (WISP2), IGFBP4 and GREB1. E2 exposure also increased expression of PDZK1, MYC, CCND1, TGFA and AREG. E2-treated MCF-7 microtissues demonstrated increased expression of SNAI1, XBP1, FOS, notch 2 (NOTCH2) and B-cell lymphoma 2 (BCL2). MCF-7 microtissues treated with 1nM E2 for 8 hours displayed decreased expression of GPER, CYP1A1 and wingless-type MMTV integration site family member 5A (WNT5A). All gene expression results are reported in S3 Table. Overlapping and unique expression changes in 2D and 3D cultures exposed to estradiol are reported in Fig 1A.

**Table 2:** 

Gene Name	Abbreviation	Fold Change	q value
Progesterone receptor	PGR	10.49	<0.0001
WNT1 Inducible Signaling Pathway Protein	WISP2	8.37	0.0126
insulin-like growth factor binding protein 4	IGFBP4	5.33	<0.0001
growth response to estrogen, breast cancer 1	GREB1	5.18	<0.0001
PDZ containing domain 1	PDZK1	5.00	<0.0001
V-Myc Avian Myelocytomatosis Viral Oncogene Homolog	MYC	3.39	<0.0001
cyclin D1	CCND1	3.31	<0.0001
transforming growth facotr alpha	TGFA	2.95	0.0002
amphiregulin	AREG	2.57	0.0012
Snail Family Zinc Finger 1,	SNAI1	2.40	0.0061
xbox binding protein 1	XBP1	2.07	0.0017
FBJ Murine Osteosarcoma Viral Oncogene Homolog	FOS	2.02	0.0049
Notch 2	NOTCH2	1.97	0.0063
B-cell lymphoma 2	BCL2	1.57	0.0431
g-protein coupled estrogen receptor	GPER	-2.14	0.0009
Cytochrome P450 1A1	CYP1A1	-3.78	<0.0001
Wingless-Type MMTV Integration Site Family, Member 5A	WNT5A	-4.26	0.0457

Monolayer MCF-7 cultures treated with 1nM E2 for 8 hours display altered expression of 21 transcripts (Table 3), with increased expression of ectonucleotide pyrophosphatase/phosphodiesterase 2 (ENPP2), PGR, AREG, GREB1, IGFBP4, PDZK1 and LRCC15 compared to vehicle-treated controls. Expression of breast cancer 1 (BRCA1), breast cancer 2 (BRCA2), cathepsin D (CTSD), BCL2, and WISP2 were significantly increased. TFF1, MYC, Aryl hydrocarbon receptor (AHR), TGFA and TGFB3 expression were significantly increased. E2 treatment of monolayer cultures for 8 hours resulted in statistically significant decreased expression of ERBB2, CYP1A1, CCND2 and WNT5A compared to vehicle-treated controls. All gene expression results following E2 exposure are included in S4 Table.

**Table 3:** 

Gene Name	Abbreviation	Fold Change	q value
Ectonucleotide Pyrophosphatase/ Phosphodiesterase 2	ENPP2	15.49	0.0063
Progesterone receptor	PGR	5.63	<0.0001
Amphiregulin	AREG	3.99	0.0026
Growth response to estrogen, breast cancer 1	GREB1	3.83	<0.0001
insulin-like growth factor binding protein 4	IGFBP4	3.33	0.0004
PDZ containg domain 1	PDZK1	3.31	<0.0001
Leucine-rich repeat containing 15	LRRC15	2.71	0.0106
Breast cancer 1	BRCA1	2.37	0.0001
Breast cancer 2	BRCA2	2.13	0.0047
Cathepsin D	CTSD	2.05	0.0002
B-cell lymphoma 2	BCL2	1.93	0.0034
WNT1 Inducible Signaling Pathway Protein	WISP2	1.92	0.0001
Trefoil factor 1	TFF1	1.80	<0.0001
V-Myc Avian Myelocytomatosis Viral Oncogene Homolog	MYC	1.72	0.0016
Aryl-hydrocarbon receptor	AHR	1.67	0.0498
Transforming growth factor alpha	TGFA	1.55	0.0052
Transforming growth factor beta 3	TGFB3	1.53	0.0108
Notch 2	NOTCH2	1.40	0.0059
Erb-B2 Receptor Tyrosine Kinase 2	ERBB2	-1.75	0.0095
Cytochrome P450 1A1	CYP1A1	-2.10	0.0001
Cyclin D2	CCND2	-2.32	0.0090
Wingless-Type MMTV Integration Site Family, Member 5A	WNT5A	-5.29	0.0001

Exposure of MCF-7 microtissues to 1nM E2 for 8 hours resulted in significantly altered expression of 20 transcripts, compared to vehicle-treated controls (Table 4). Estradiol induced expression of PGR, GREB1, IGFBP4, PDZK1, TGFB3 and MYC. Expression of TGFA, SNAI1, CTSD, FOS, BRCA1 and XBP1 were significantly increased by E2 treatment. Complete gene expression results can be found in S5 Table. Differences and similarities in expression are summarized in Fig 1B.

**Table 4:** 

Gene Name	Abbreviation	Fold Change	q value
Progesterone receptor	PGR	8.52	<0.0001
growth response to estrogen, breast cancer 1	GREB1	7.05	<0.0001
insulin-like growth factor binding protein 4	IGFBP4	6.10	<0.0001
PDZ containing domain 1	PDZK1	5.43	<0.0001
Transforming growth factor beta 3	TGFB3	3.09	<0.0001
V-Myc Avian Myelocytomatosis Viral Oncogene Homolog	MYC	2.99	<0.0001
transforming growth factor alpha	TGFA	2.38	<0.0001
Snail Family Zinc Finger 1,	SNAI1	1.99	0.0006
Cathepsin D	CTSD	1.88	0.0004
FBJ Murine Osteosarcoma Viral Oncogene Homolog	FOS	1.88	0.0002
Breast cancer 1	BRCA1	1.72	0.0031
xbox binding protein 1	XBP1	1.62	0.0003
Estrogen receptor alpha	ESR1	-1.50	0.0031
Twist Family BHLH Transcription Factor 1	TWIST1	-1.70	0.0021
g-protein coupled receptor 1	GPER	-1.71	0.0006
Matrix metallopeptidase 9	MMP9	-1.75	0.0001
Erb-B2 Receptor Tyrosine Kinase 2	ERBB2	-2.05	0.0020
Cytochrome P450 1A1	CYP1A1	-2.22	<0.0001
Cyclin D2	CCND2	-2.86	0.0026
Brain-derived neurotrophic factor	BDNF	-4.34	0.0001

MCF-7 monolayers exposed to 1nM E2 for 24 hours displayed increased expression of 5 transcripts (Table 5), including IGFBP4, PGR, PDZK1, AREG and MYBL2. Complete results are reported in S6 Table.

**Table 5:** 

Gene Name	Abbreviation	Fold Change	q value
Insulin-Like Growth Factor Binding Protein 4	IGFBP4	3.68	0.0002
Progesterone Receptor	PGR	6.98	0.0017
PDZ domain containing 1	PDZK1	7.27	0.0030
Amphiregulin	AREG	3.84	0.0184
V-Myb Avian Myeloblastosis Viral Oncogene Homolog-Like 2	MYBL2	2.47	0.0468

Exposure to E2 for 24 hours significantly altered expression of 22 transcripts in MCF-7 microtissues (Table 6). Expression of BCL2-like 2 (BCL2L2), cystatin D (CST5), PDZK1, AREG, leucine rich repeat containing 15 (LRRC15) and IGFBP4 was significantly increased following estradiol exposure. CTSD, PGR, prostaglandin synthase 2 (PTGS2), snail family zinc finger 2 (SNAI2) and claudin 1 (CLDN1) expression was significantly increased in MCF-7 microtissues treated with E2 for 24 hours. Increases in expression of TGFB3, MYC, cyclin A1 (CCNA1), MYBL2, TGFA, N-cadherin (CDH2), fibronectin (FN1), BRCA1 and FOS were observed in E2-treated microtissues (Table 6). Expression of gap junction 1 (GJA1) and integrin alpha 1 (ITGA1) was significantly decreased in E2-treated microtissues. Complete gene expression results are reported in S7 Table. Monolayer and microtissue cultures treated with E2 for 24 hours displayed large differences in gene expression (Fig 1C).

**Table 6:** 

Gene Name	Abbreviation	Fold Change	q value
Insulin-Like Growth Factor Binding Protein 4	IGFBP4	3.35	0.0002
PDZ domain containing 1	PDZK1	4.49	0.0004
Amphiregulin	AREG	4.01	0.0006
Progesterone Receptor	PGR	3.08	0.0010
Transforming growth factor beta 3	TGFB3	2.35	0.0015
V-Myc Avian Myelocytomatosis Viral Oncogene Homolog	MYC	2.05	0.0026
Claudin 1	CLDN1	2.54	0.0042
V-Myb Avian Myeloblastosis Viral Oncogene Homolog-Like 2	MYBL2	1.87	0.0049
transforming growth factor alpha	TGFA	1.84	0.0070
Snail Family Zinc Finger 2	SNAI2	2.85	0.0072
Leucine rich repeat containing 15	LRRC15	3.66	0.0073
Gap Junction 1	GJA1	-2.18	0.0079
Cyclin A1	CCNA1	2.02	0.0081
Breast cancer 1	BRCA1	1.65	0.0084
Fibronectin 1	FN1	1.66	0.0103
FBJ Murine Osteosarcoma Viral Oncogene Homolog	FOS	1.55	0.0105
Prostaglandin synthase 2	PTGS2	2.97	0.0117
Integrin, alpha 1	ITGA1	4.07	0.0147
BCL-like 2	BCL2L2	34.16	0.0192
Cathepsin D	CTSD	3.25	0.0194
Cystatin D	CST5	6.95	0.0197
N-cadherin	CDH2	1.82	0.0311

## Discussion

When compared to 2D monolayer MCF-7 cultures, MCF-7 microtissues display differences in gene expression following exposure to estradiol. The current study indicates that in both 2D and 3D cultures exposed to estradiol, genes that are typically used as indicators of estrogenic response are upregulated by estradiol. These genes include PGR, PDZK1 [22], and IGFBP4 [23, 24], which are all increased at 4, 8, and 24 hours of exposure in 2D and 3D cultures, though the degree of fold change is greater in 3D microtissues. The similar induction of transcription of other well-known estrogen responsive genes including GREB1 [22], AREG [25], MYC and inhibition of CYP1A1 demonstrates that in terms of estrogenic activity, MCF-7 monolayers and 3D microtissues respond similarly. Following hierarchical clustering analysis, these genes cluster together and exhibit similar patterns in 2D and 3D cultures over time (Fig 1D). The primary response encompasses changes in expression of genes that contain estrogen response elements, and is consistent with previous studies that demonstrate the binding of ERs and transcription of target genes following exposure to estradiol [22].

Interestingly, transcription of other genes in 2D and 3D MCF-7 cultures is not similar following E2 exposure. As early as 4 hours of E2 exposure there are differences in expression of many markers between 2D and 3D cultures, though the majority of significantly altered genes are changed in both 2D and 3D cultures exposed to E2. Over time, the number of genes that are significantly altered in 2D or 3D cultures increases, while the number of genes shared between the two decreases, indicating a divergence of the response in each system. Hierarchical clustering demonstrated that genes expressed in 3D microtissues and not in 2D cluster separately from typical estrogen-responsive genes (Fig 1D). Expression of downstream genes involved in cell cycle progression, cell-cell communication and cell adhesion is differentially altered in monolayer and 3D cultures after 24 hours of exposure. The large differences in expression of cell adhesion markers at later time points is likely due to the differences in cellular differentiation between 2D and 3D cultures that have been previously described [15]. This difference is most striking at 24 hours of estradiol exposure, where 2D cultures display changes in only 5 genes, while 3D cultures have 22 transcripts significantly changed. A number of the 22 genes altered by estradiol exposure in 3D cultures are involved with cell adhesion (FN1, ITGA1, CST5, CDH2, CLDN1, SNAI2 and GJA1) These changes in cell adhesion molecules were not detected in E2-treated monolayers in the present study. *In* vitro and *in vivo* studies have demonstrated that exposure to E2 increases epithelial-mesenchymal transition (EMT), increasing the invasive potential and mobility of cells [26, 27]. In the present study, increases in expression of the EMT transcriptional regulators TWIST1 [28] and SNAI1 [29] are detected as early as 8 hours. Both SNAI1 and TWIST initiate EMT, which results in degradation of cell-cell contacts, loss of cellular polarity and increased invasive potential [30]. Following those increases in expression at 8 hours, at 24 hours increased expression of several classical EMT markers was detected, including FN1, CLDN1 and SNAI2. Previous work has demonstrated that CLDN1 expression is capable of inducing EMT [31], while FN1 and SNAI2 are highly expressed in mesenchymal-like cells [32, 33]. Coupled with the increased expression of mesenchymal markers was the decreased expression of epithelial markers ITGA1 and GJA1 in MCF-7 microtissues treated with E2 for 24 hours, indicating that MCF-7 cells cultured in 3D begin to undergo EMT. Interestingly, in this study, MCF-7 monolayers did not demonstrate any significant changes in expression of EMT-related genes following E2 exposure. It is clear from the current study that 2D and 3D MCF-7 cultures respond differently to E2, underlining the importance of selecting a relevant cell system.

**Fig 2:**
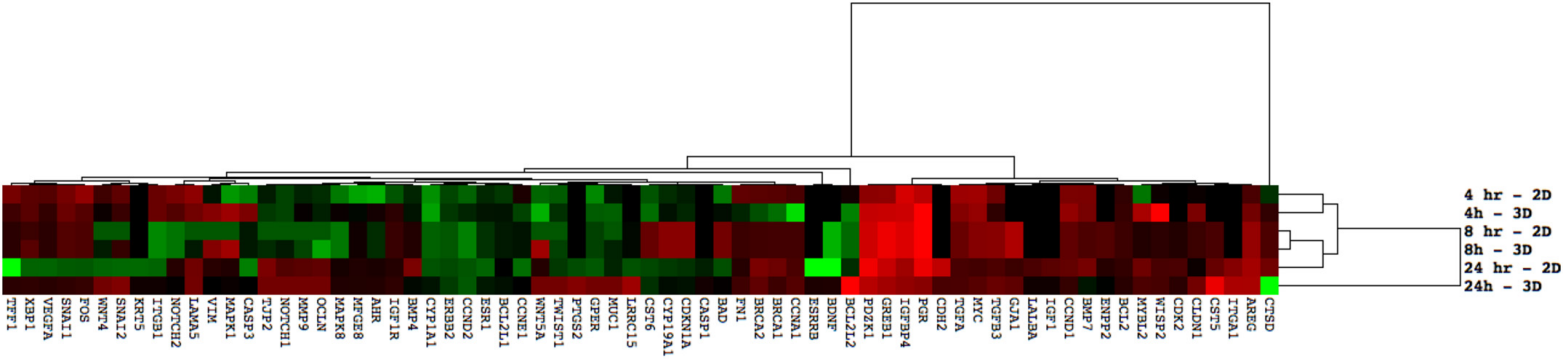
Euclidean hierarchical clustering of all significantly altered genes over time. All fold change values for significantly changed transcripts were analyzed using hierarchical clustering. Transcripts clustered into 2 large groups, aligning with the observation that classical estrogen responsive genes (ex PGR, GREB, AREG) are similarly expressed in 2D and 3D cultures, but secondary responses are different (ex VIM, OCLN). Green is decreased expression, red indicates increased expression.

Overall, the current study indicates that when examining 2D and 3D cultures for estrogenic responses by assessing the expression of target genes, levels of expression of classical estrogen-induced genes are similar at early time points. However, at later time points, the estrogenic responses diverge and result in activation of different targets in each culture system (Fig 2). In this analysis, both 2D and 3D 4 hour cultures clustered together, with the 8 hour 2D, 8 hour 3D and 24 hour 2D samples clustering together. The 24 hour 3D sample clustered separately from all treatments, further emphasizing the large differences observed in 2D and 3D culture systems. Expression of downstream genes involved in cell cycle progression, cell-cell communication and cell adhesion are significantly altered in 3D cultures and not 2D cultures. The large differences in expression of cell adhesion markers at later time points is likely due to the differences in cellular differentiation between 2D and 3D cultures that have been previously described [15]. This observation further supports the need to develop more physiologically relevant, human cell based models for toxicity testing, as underlying differences in gene and protein expression in various systems can lead to altered interpretation of results.

## Supporting Information

S1 Table Genes included on custom PCR array. (XLS)

S2 Table Complete gene expression results for MCF-7 monolayer cultures treated with 1nM estradiol for 4 hours. Values determined relative to vehicle-treated controls, positive fold change indicates increased expression.
(XLSX)

S3 Table MCF-7 microtissues exposed to 1nM estradiol for 4 hours demonstrate alterations in gene expression. Positive fold change indicates increased expression, values compared to time-matched, vehicle-treated controls.
(XLSX)

S4 Table Monolayer MCF-7 cultures have altered gene expression following 8 hours of exposure to 1nM estradiol. Positive fold change indicates increased expression, relative to vehicle-treated controls.
(XLSX)

S5 Table MCF-7 microtissues demonstrate altered gene expression following exposure to 1nM estradiol for 8 hours. Fold change relative to vehicle-treated controls.
(XLSX)

S6 Table Complete gene expression results of monolayer MCF-7 cultures exposed to estradiol for 24 hours. Fold change values relative to vehicle-treated controls.
(XLSX)

S7 Table Complete gene expression results for MCF-7 microtissues exposed to 1nM estradiol for 24 hours. Fold change relative to vehicle-treated controls.
(XLSX)
